# Supplementary material for: Effects of Gut Microbiome Modulation on Reducing Adverse Health Outcomes among Elderly and Diabetes Patients during the COVID-19 Pandemic: A Randomised, Double-Blind, Placebo-Controlled Trial (IMPACT Study)
Source: Nutrients. 2023 Apr 20;15(8):1982. doi: 10.3390/nu15081982 (PMC10143994; doi:10.3390/nu15081982)
Supplement: Supplementary file 1 [file nutrients-15-01982-s001.zip › nutrients-2342777-supplementary.pdf]

Table S1. Grouping of pathogenic bacteria according to the faecal metagenomic analysis of microbial profile

**Species**	**Group**	**Publication**
s__Bifidobacterium_adolescentis	Covid_Negative_Cor	
s__Bifidobacterium_pseudocatenulatum	Covid_Negative_Cor	
s__Collinsella_aerofaciens	Covid_Negative_Cor	
s__Bacteroides_caccae	Covid_Positive_Cor	
s__Bacteroides_dorei	Covid_Positive_Cor	
s__Bacteroides_ovatus	Covid_Positive_Cor	
s__Bacteroides_vulgatus	Covid_Positive_Cor	
s__Blautia_obeum	Covid_Negative_Cor	
s__Ruminococcus_gnavus	Covid_Positive_Cor	Yeoh Y K, et al. Gut, 2021, 70(4): 698-706.
s__Ruminococcus_torques	Covid_Positive_Cor	
s__Coprococcus_comes	Covid_Negative_Cor	
s__Dorea_formicigenerans	Covid_Negative_Cor	
s__Dorea_longicatena	Covid_Negative_Cor	
s__Eubacterium_rectale	Covid_Negative_Cor	
s__Faecalibacterium_prausnitzii	Covid_Negative_Cor	
s__Ruminococcus_bromii	Covid_Negative_Cor	
s__Ruminococcus_obeum_CAG_39	Covid_Negative_Cor	
s__Akkermansia_muciniphila	Covid_Positive_Cor	
s__Bacteroides_fragilis	Long_covid_Positive_Cor	
s__Bacteroides_salyersiae	Long_covid_Positive_Cor	
s__Blautia_hansenii	Long_covid_Positive_Cor	
s__Blautia_sp_CAG_257	Long_covid_Positive_Cor	
s__Ruminococcus_gnavus	Long_covid_Positive_Cor	
s__Clostridium_bolteae	Long_covid_Positive_Cor	
s__Coprobacillus_cateniformis	Long_covid_Positive_Cor	
s__Erysipelatoclostridium_ramosum	Long_covid_Positive_Cor	
s__Parasutterella_excrementihominis	Long_covid_Positive_Cor	
s__Sutterella_parvirubra	Long_covid_Positive_Cor	Su Q, Lau R I, Liu Q, et al.. Gut, 2022.
s__Bifidobacterium_adolescentis	Long_covid_Negative_Cor	
s__Bifidobacterium_pseudocatenulatum	Long_covid_Negative_Cor	
s__Bacteroides_dorei	Long_covid_Negative_Cor	
s__Eubacterium_hallii	Long_covid_Negative_Cor	
s__Blautia_obeum	Long_covid_Negative_Cor	
s__Blautia_wexlerae	Long_covid_Negative_Cor	
s__Roseburia_hominis	Long_covid_Negative_Cor	
s__Gemmiger_formicilis	Long_covid_Negative_Cor	
s__Firmicutes_bacterium_CAG_83	Long_covid_Negative_Cor	
s__Megamonas_funiformis	Long_covid_Negative_Cor	

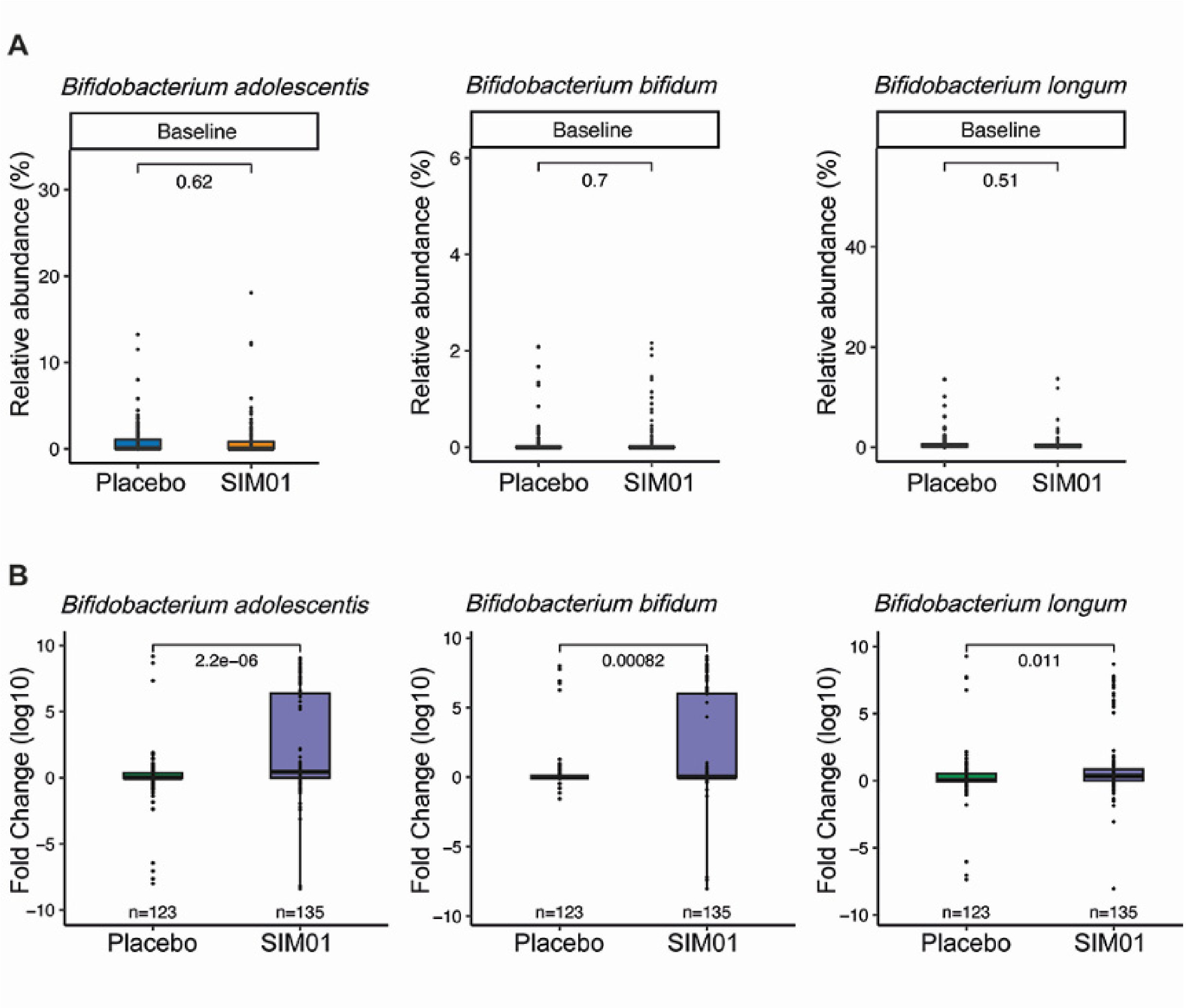

Supplementary Figure S1. (A) There is no difference in the relative abundance of three species (*Bifidobacterium adolescentis*, *Bifidobacterium bididum*, and *Bifidobacterium longum*) contained in SIM01 between the placebo and SIM01 groups at baseline. (B) Oral SIM01 treatment significant increase in the fold change (month 3/baseline) of three probiotic species contained in SIM01 compared with the placebo arm. *p* values were given by the Wilcoxon rank-sum test (two-sided). Elements on boxplots: centre line, median; box limits, upper and lower quartiles; points, each sample.
